# Alteration of active and repressive histone marks during adipogenic differentiation of porcine mesenchymal stem cells

**DOI:** 10.1038/s41598-020-79384-x

**Published:** 2021-01-14

**Authors:** Joanna Stachecka, Pawel A. Kolodziejski, Magdalena Noak, Izabela Szczerbal

**Affiliations:** 1 Department of Genetics and Animal Breeding, Poznan University of Life Sciences, Wolynska 33, 60-637 Poznan, Poland; 2 Department of Animal Physiology, Biochemistry and Biostructure, Poznan University of Life Sciences, Wolynska 35, 60-637 Poznan, Poland

**Keywords:** Nuclear organization, Epigenetics, Mesenchymal stem cells

## Abstract

A characteristic spatial distribution of the main chromatin fractions is observed in most mammalian cell nuclei, with euchromatin localized in the interior and heterochromatin at the nuclear periphery. It has been shown that interactions of heterochromatin with the nuclear lamina are necessary to establish this conventional architecture. Adipocytes are specific cells in which a reduction in lamin A/C expression is observed. We hypothesize that the loss of lamin A/C during adipogenic differentiation of mesenchymal stem cells (MSCs) may be associated with the reorganization of the main classes of chromatin in the nucleus. Thus, in this study, we examine the abundance and nuclear distribution of selected heterochromatin (H3K9me3, H3K27me3 and H4K20me3) and euchromatin (H4K8ac, H3K4me3 and H3K9ac) histone marks during in vitro adipogenesis, using the pig as a model organism. We found that not only did the expression of lamin A/C decrease in our differentiation system, but so did the expression of lamin B receptor (LBR). The level of two heterochromatin marks, H3K27me3 and H4K20me3, increased during differentiation, while no changes were observed for H3K9me3. The levels of two euchromatin histone marks, H4K8ac and H3K9ac, were significantly higher in adipocytes than in undifferentiated cells, while the level of H3K4me3 did not change significantly. The spatial distribution of all the examined histone marks altered during in vitro adipogenesis. H3K27me3 and H4K20me3 moved towards the nuclear periphery and H3K9me3 localized preferentially in the intermediate part of adipocyte nuclei. The euchromatin marks H3K9ac and H3K4me3 preferentially occupied the peripheral part of the adipocyte nuclei, while H4K8ac was more evenly distributed in the nuclei of undifferentiated and differentiated cells. Analysis of the nuclear distribution of repetitive sequences has shown their clustering and relocalization toward nuclear periphery during differentiation. Our study shows that dynamic changes in the abundance and nuclear distribution of active and repressive histone marks take place during adipocyte differentiation. Nuclear reorganization of heterochromatin histone marks may allow the maintenance of the nuclear morphology of the adipocytes, in which reduced expression of lamin A/C and LBR is observed.

## Introduction

Adipogenesis, the process of fat cell formation, is controlled on both transcriptional and epigenetic levels. A number of proadipogenic and antiadipogenic transcription factors have been identified^1^, and epigenetic mechanisms—such as DNA methylation, histone modification, and chromatin remodeling—have been found to play a role in the process^2^. The differentiation of progenitor cells into adipocytes is accompanied not only by characteristic changes in cell morphology (resulting in round lipid-laden cells with a rim of cytoplasm and a flattened peripherally located nucleus), but also in nuclear organization^3,4^. This includes changes in nucleus shape and size, reduction of lamin A/C expression, reorganization of the chromocenter, and repositioning of chromosomes and genes^4–8^. Moreover, higher-order chromatin organization and chromatin interactions have recently emerged as important regulators of gene expression during adipogenesis^9–11^.

The chromatin in the mammalian cell nucleus is organized hierarchically. Two compartments, A and B, have been distinguished, representing transcriptionally active and repressive environments, respectively^12^. In conventional nucleus types, the euchromatin is localized in the interior and the heterochromatin is located at the nuclear periphery^13^. Inverted organization of the main chromatin fractions is observed in rod cells of nocturnal animals, where heterochromatin localizes in the nuclear center and euchromatin on the nuclear border^14^. These nuclei are lacking in lamin A/C and lamin B receptor (LBR)^15^. It has been shown that attractions between heterochromatic regions are crucial for establishing inverted nuclear architecture, and interactions of heterochromatin with the nuclear lamina are needed to build the conventional architecture^16^. It is assumed that the segregation of chromatin into active and silent nuclear compartments has functional significance^17^.

Adipocytes are specific cells in which reduction of lamin A/C expression is observed. The drop of lamin A/C abundance during adipogenesis affects the mechanical properties of nuclei, which become less stiff and more resilient to the pressure of the enlarging lipid droplets that fill the cellular space of adipocytes^18^. Studies of cytoskeletal and nuclear stiffness of MSCs have shown that the generation of adipocytes (which form a soft tissue) is associated with increased LBR expression levels and low lamin A/C levels, unlike in the development of bone (a stiff tissue), characterized by a high level of lamin A/C^19,20^. Lamin A/C is also involved in a WNT/β-catenin metabolic pathway that directs mesenchymal stem cells towards osteogenic differentiation. Decreases in lamin A/C expression are thus important for the suppression of osteogenic differentiation while facilitating adipogenic differentiation of mesenchymal stem cells (MSC) in vitro and in vivo^21^. Nuclear lamins play a role in gene regulation, since most genes in lamina-associated domains (LADs) are repressed or expressed at very low level^22^. Many examples of the genes encoding key transcription factors of adipogenesis being repositioned from the nuclear periphery to the nuclear center have been described during adipogenesis^6,8,23^.

Transcriptionally active and repressed chromatin possesses characteristic histone modifications^24^. Heterochromatin is marked by H3K27me3, H3K9me2/3, and H4K20me3, while modifications such as H3K9ac, H3K4me3, H3K27ac, and H3K36me3 mark euchromatin^17,25^. Changes in specific histone modification levels occur in differentiation processes, including the differentiation of MSCs^26,27^. It has been shown that MSCs differentiating into adipocytes have a significantly different histone pattern than undifferentiated MSCs, which was not observed for osteogenic differentiation^28^. It is believed that dynamics in histone modifications are important for MSC lineage commitment^29^. Most studies of histone modification during MSC differentiation into adipocytes have focused on the distribution of these modifications in specific genomic regions or specific genes^28,30,31^. However, little is known about changes in the radial distribution of histone marks and the main classes of chromatin in cell nuclei during MSC differentiation.

We hypothesize here that MSCs undergo significant changes in global reorganization of main chromatin classes during adipogenic differentiation, as a result of the reduction in lamin A/C expression. We thus examined the abundance and nuclear distribution of selected heterochromatin (H3K9me3, H3K27me3 and H4K20me3) and euchromatin (H4K8ac, H3K9ac, and H3K4me3) histone marks, as well as the three-dimensional nuclear location of repetitive sequences during in vitro adipogenesis, using a pig as a model organism.

## Results

### Adipogenic differentiation and expression of nuclear envelope proteins

**Figure 1:**
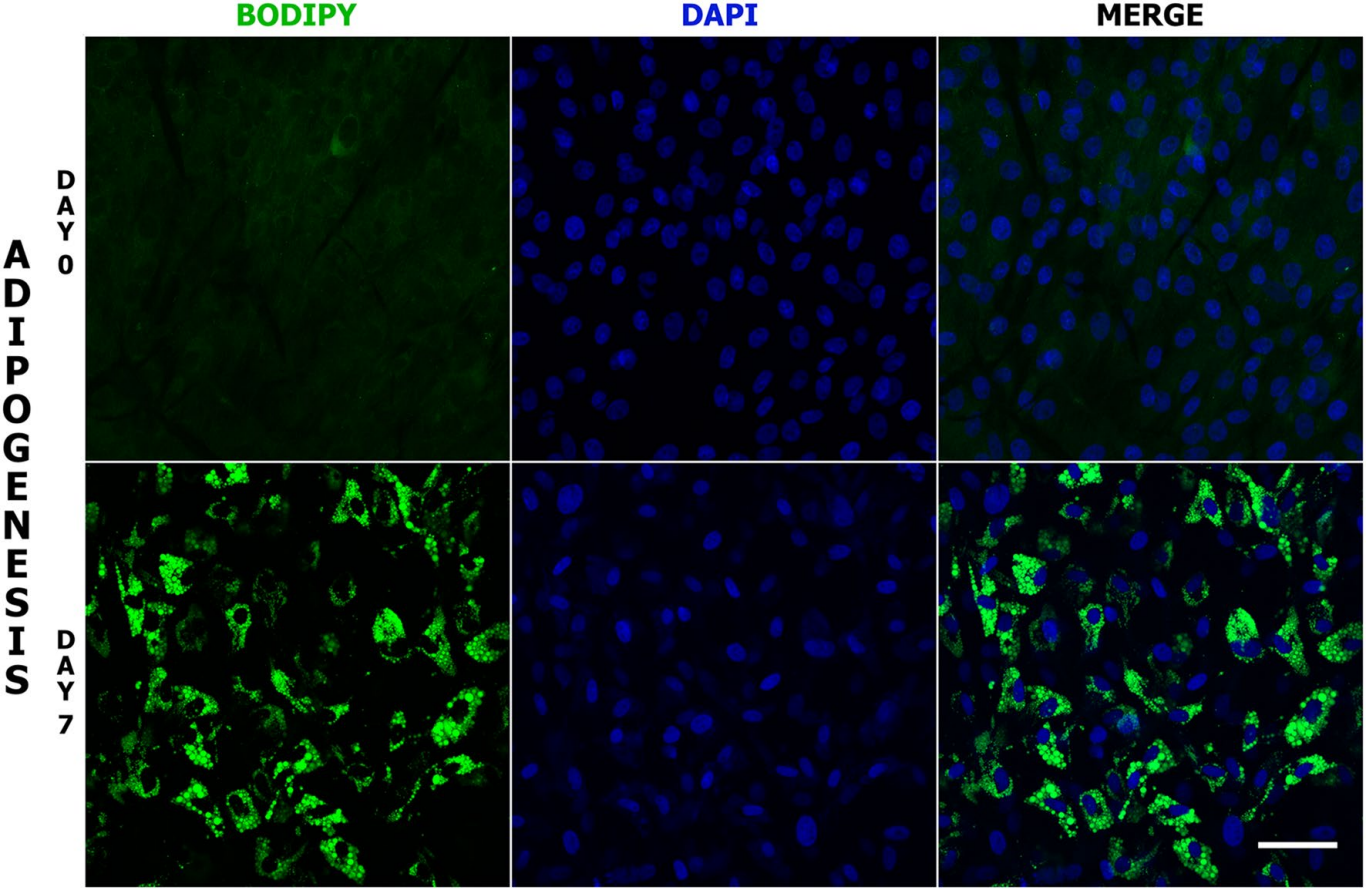
Monitoring of lipid droplet formation during in vitro adipogenesis. Visualization of lipid droplets in AD-MSC on days 0 and 7 of adipogenic differentiation. The droplets were stained with BODIPY (green) while the nuclei were counterstained with DAPI (blue). Scale bar: 50 µm.

**Figure 2:**
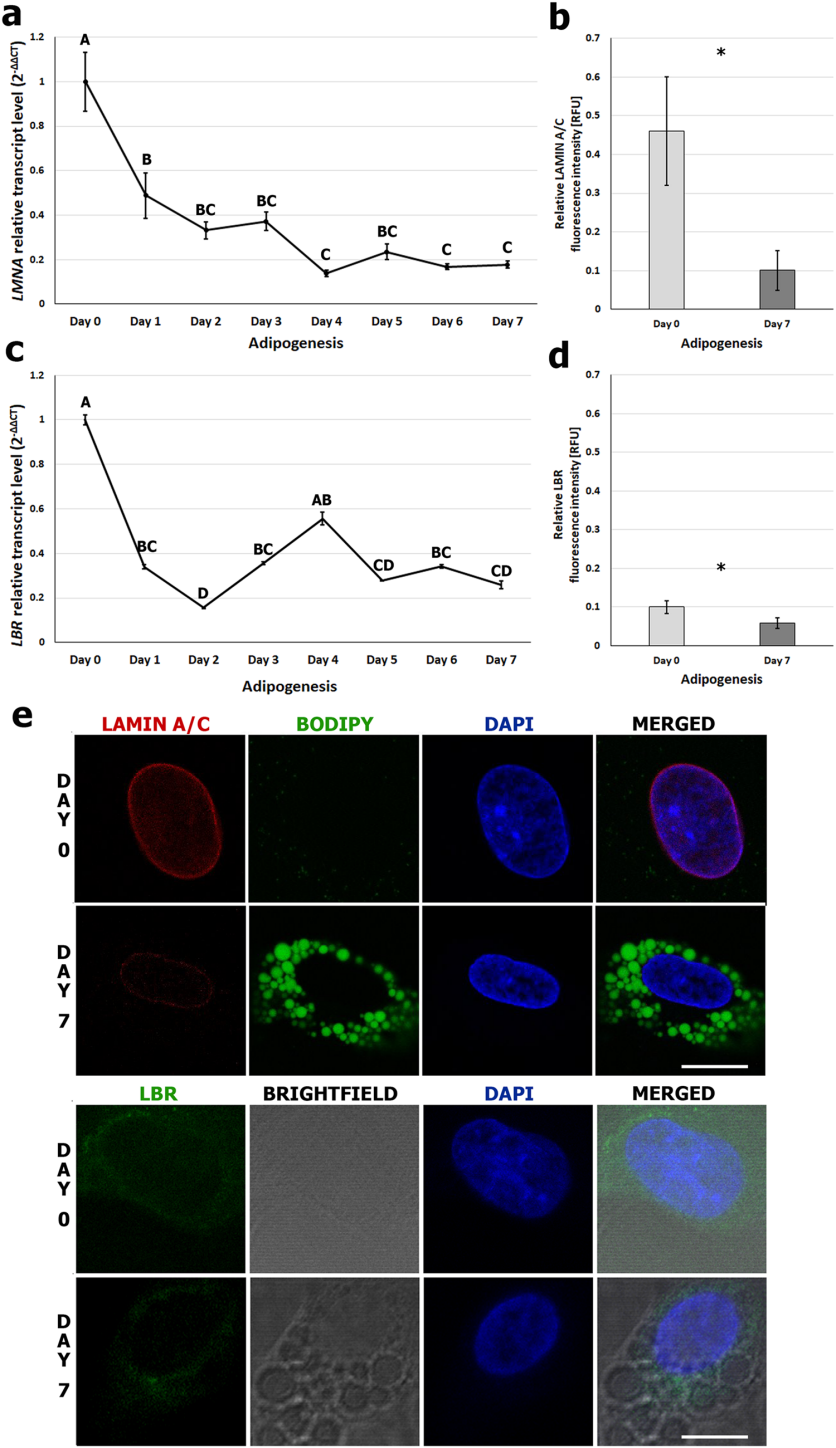
Expression of lamin A/C and lamin B receptor during porcine in vitro adipogenesis. (**a**) Relative transcript levels of the *LMNA* gene over seven days of differentiation. Different capital letters refer to significant differences between days (*P* < 0.05); error bars show SD. (**b**) Comparison of immunofluorescent signal intensities of lamin A/C normalized by DAPI fluorescence intensity signal on days 0 and 7 of in vitro adipogenesis; error bars show SD. Statistically significant differences (*P* < 0.05) are marked with asterisks. The number of nuclei analyzed was n = 169. (**c**) Relative transcript levels of *LBR* gene on subsequent days of adipogenic differentiation. Different capital letters refer to significant differences between analyzed days (*P* < 0.05); error bars show SD. (**d**) Comparison of immunofluorescent signal intensities of LBR normalized by DAPI fluorescence intensity on days 0 and 7 of in vitro adipogenesis; error bars shows SD. Statistically significant differences (*P* < 0.05) are marked with asterisks. The number of nuclei analyzed was n = 280. (**e**) Visualization of lamin A/C (red) or LBR (green) by indirect immunofluorescent staining in undifferentiated MSC cells (day 0) and adipocytes (day 7). Lipid droplets were stained using BODIPY (green) or visualized in brightfield and nuclei were counterstained with DAPI (blue). Scale bar: 10 µm.

A system of in vitro differentiation of MSC derived from adipose tissue (AD-MSC) into adipocytes was used. This system has exhibited strong adipogenic potential, as assessed by the accumulation of lipid droplets; the percentage of cells containing lipid droplets on day 7 of adipogenesis was 71% (Fig. 1). In the first step of the study, the expression of lamin A/C was evaluated in undifferentiated cells (day 0) and over the seven-day process of differentiation. The transcript of the *LMNA* gene was detected in each day of adipogenesis; however, a significant decrease in its level was seen on day 1 (49.48% of initial transcript level) (Fig. 2a). In the following days of adipogenesis, the *LMNA* transcript level was stable and low (37.43%–13.91% of the initial level). In the absence of lamin A/C, lamin B receptor is known to act as a scaffold tethering the spatial distribution of heterochromatin and euchromatin. For this reason, we next examined the transcript levels of the *LBR* gene, finding that it decreased during adipogenic differentiation, reaching its lowest point on day 2 (15.65% of the initial level) and exhibit a small peak on day 4 (55.58% of initial level) (Fig. 2c). On day 7 of adipogenesis, the decrease in relative transcript level was greater for the *LMNA* gene than for *LBR* (17.85% of the initial level (*P* = 0.03) versus 25.92% of the initial level (*P* = 0.004) (Fig. 2a,c). The initial transcript level of *LMNA* was higher than *LBR* (0.29 for *LMNA* versus 0.0059 for *LBR* analyzed in relation to a reference gene) (Supplementary Fig. S1).

The downregulation of lamins was also observed on the protein level. An immunofluorescent staining on cultured MSC and adipocytes confirmed the loss in lamin A/C abundance (Fig. 2b,e). Analysis of relative fluorescence signal intensities showed that lamin A/C content decreased by 90.95% when comparing undifferentiated cells and adipocytes on day 7 (*P* < 0.00001) (Fig. 2b). Immunofluorescent staining with antibody against LBR gave very weak signals on cultured MSC and in adipocytes (Fig. 2d,e; Supplementary Fig. S2). Analysis of relative fluorescence signal intensities showed that the level of LBR on day 7 decreased by 41.54% (*P* < 0.00001) compared to undifferentiated cells (Fig. 2d).

To confirm that decreased expression of the two studied nuclear envelope proteins is typical of adipocyte differentiation, we compared the transcript level of *LMNA* and *LBR* during osteogenic and chondrogenic differentiations (Supplementary Fig. S3). The *LMNA* increased during osteogenic and chondrogenic differentiations, while the relative transcript level of *LBR* decreased in all differentiation systems, reaching its lowest value during adipogenesis (*P* < 0.05).

### Changes in abundance of heterochromatin and euchromatin histone marks in MSC and adipocytes

**Figure 3:**
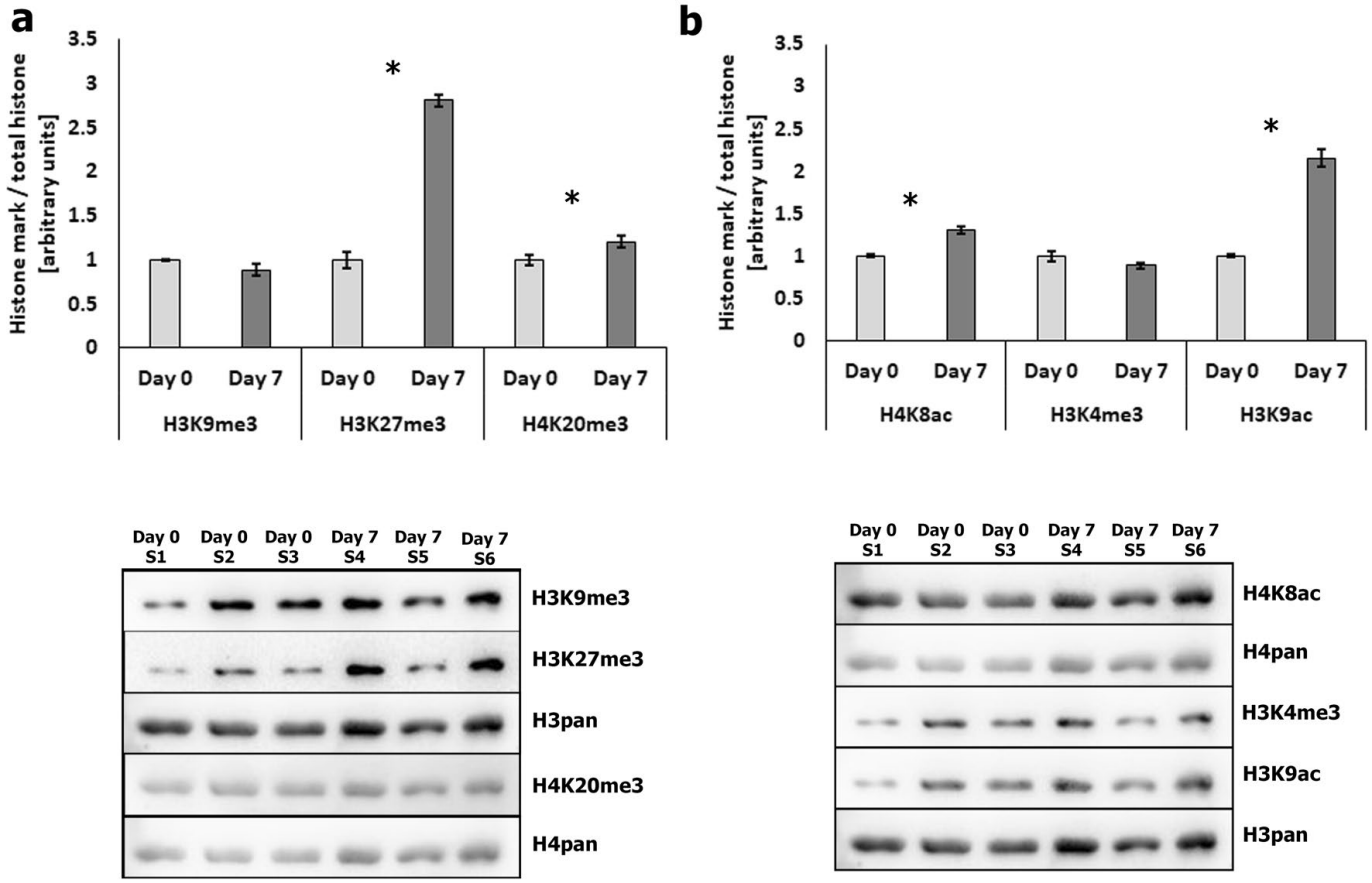
Western blot analysis of histone modification levels during adipogenesis. (**a**) The abundance of H3K9me3, H3K27me3, and H4K20me3 modifications in undifferentiated cells (day 0) and adipocytes (day 7). Statistically significant differences (*P* < 0.05) are marked with asterisks. (**b**) Abundance of H4K8ac, H3K4me3, and H3K9ac histone modifications in undifferentiated cells (day 0) and adipocytes (day 7). Statistically significant differences (*P* < 0.05) are marked with asterisks. Full length blots are presented in Supplementary Figures S4 and S5.

Since lamin A/C and LBR are involved in maintaining the spatial distribution of the main chromatin fractions in conventional nuclei, we next looked at the abundance of selected histone marks characteristic of heterochromatin (H3K9me3, H3K27me3, and H4K20me3) and euchromatin (H4K8ac, H3K4me3, and H3K9ac). Protein samples isolated form undifferentiated MSCs (day 0) and adipocytes (day 7) were analyzed using western blot (Fig. 3, Supplementary Fig. S4, S5). We found that two heterochromatin marks increased significantly in the adipocytes at day 7, compared to undifferentiated: H3K27me3 (increased by a factor of 2.81; *P* < 0.0001) and H4K20me3 (increased by a factor of 1.30; *P* = 0.019) (Fig. 3a). The level of H3K9me3 remained stable during differentiation. Among euchromatin-specific histone modifications, significant differences were observed for H4K8ac (increased by a factor of 1.3; *P* = 0.006) and H3K9ac (increased by a factor of 2.16; *P* = 0.008) (Fig. 3b). No changes were found in the H3K4me3 level. We also compared the abundance of the modifications by measuring the relative fluorescence intensity on confocal images of immunofluorescent staining of cells fixed at days 0 and 7 of adipogenic differentiation (Supplementary Fig. S6), finding that the relative fluorescence intensities of all the heterochromatin histone marks increased during adipocyte differentiation: H3K9me3 increased by a factor of 1.28 (*P* < 0.0001), H3K27me3 increased by a factor of 4.13 (*P* < 0.0001) and H4K20me3 increased by a factor of 1.53 (*P* < 0.0001). The results obtained from the measurements of fluorescence intensities were in agreement with the western blot results for H3K27me3 and H4K20me3. The abundance of two other euchromatin histone marks, measured as relative fluorescence intensity, decreased during adipogenesis: H3K4me3 decreased by a factor of 0.46 (*P* < 0.0001) and H3K9ac by a factor of 0.68 (*P* < 0.0001). No significant changes were found when comparing the fluorescence intensities of H4H8ac modification at days 0 and 7 of adipogenesis. The abundance of euchromatin histone marks, as determined by relative fluorescence intensities, varied from the results obtained with western blot assay, which could be due to the lower precision of the immunofluorescence method.

### Spatial distribution of heterochromatin histone marks in MSC and adipocytes

**Figure 4:**
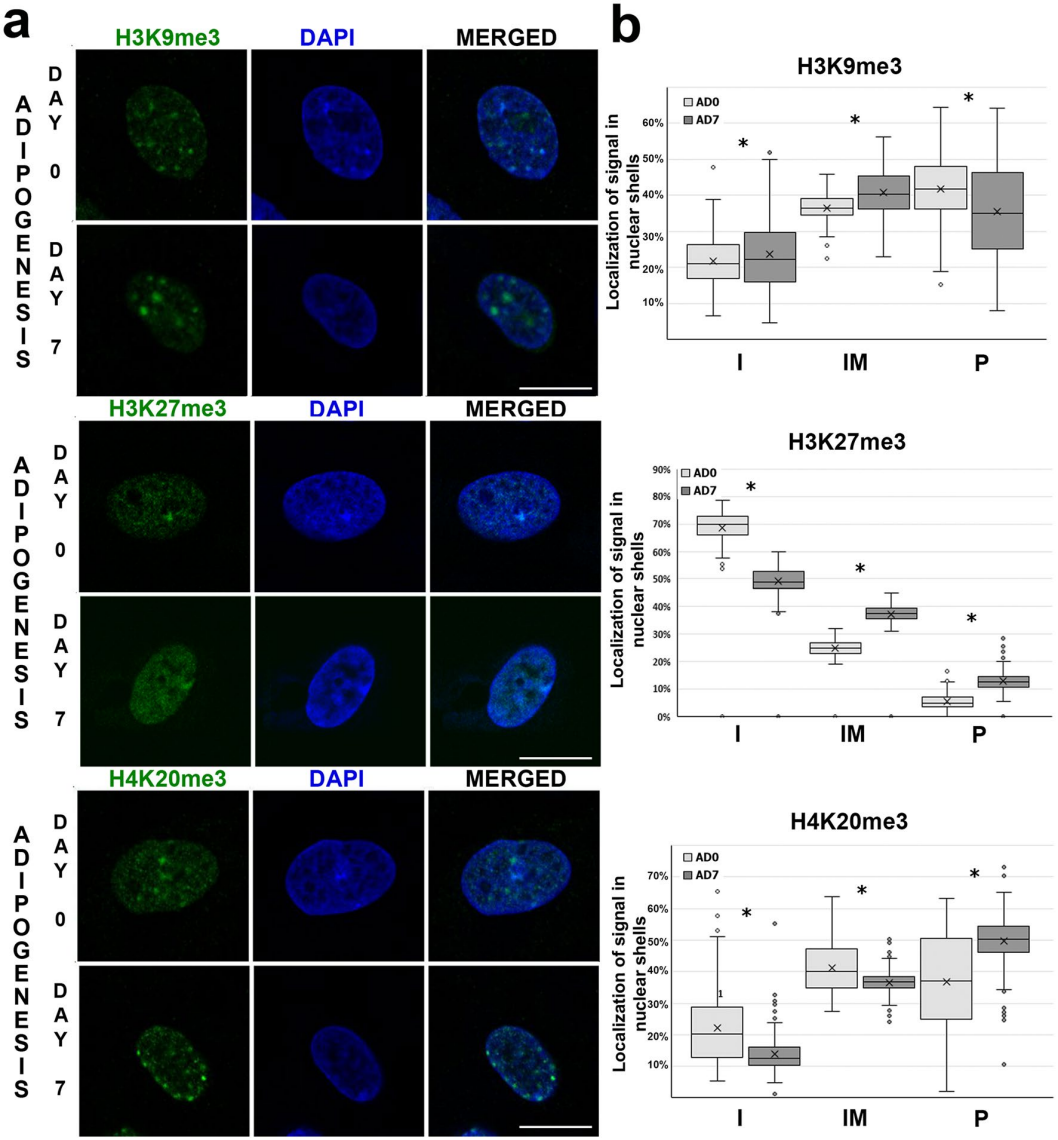
Spatial distribution of selected histone marks associated with heterochromatin. (**a**) Distribution of H3K9me3, H3K27me3, and H4K20me3 in a 3D nuclear space at days 0 and 7 of in vitro adipogenesis. The histone marks were visualized by indirect immunofluorescence (green). Nuclei were counterstained with DAPI (blue). Scale bar: 10 µm. (**b**) The graphs show spatial distribution of immunofluorescent signals in 3D nuclear space. Statistically significant differences (*P* < 0.05) are marked with asterisks. Number of nuclei analyzed: H3K9me3: n = 497, H3K27me3: n = 243, H4K20me3: n = 604. Statistical data are shown in Supplementary Table 1.

The spatial distribution of selected heterochromatin marks was determined by detection of immunofluorescent signals in the three dimensional nuclear space. For this purpose nuclei were divided into three shells, referred to as nuclear interior (I), intermediate (IM), and periphery (*P*) (Supplementary Fig. S7). It was found that H3K9me3 modification was preferentially localized in intermediate and peripheral parts of nuclei, rather than in the nuclear interior (*P* < 0.05). During in vitro adipogenesis, the distribution of this mark shifted towards the intermediate part of the nuclei. The presence of the H3K9me3 mark in the peripheral nuclear shell was less abundant in adipocytes (day 7) than in undifferentiated cells (day 0) (*P* < 0.0001) (Fig. 4b). The signals specific to the H3K9me3 mark formed distinctive foci, often located at the nuclear periphery and in proximity to the nucleoli (Fig. 4a). The H3K27me3 histone mark occurred mostly in the nuclear interior of nuclei of both undifferentiated cells and adipocytes. When nuclei at day 0 of adipogenesis were compared with nuclei at day 7 of adipogenesis, the proportion of signals in the intermediate (*P* < 0.0001) and peripheral (*P* < 0.0001) shells was seen to have significantly increased (Fig. 4b). Analysis of nuclear distribution H4K20me3 histone mark showed that in both nuclei of undifferentiated cells and nuclei on day 7 of adipogenic differentiation, this modification was preferentially localized in the intermediate and peripheral part of the nuclei (Fig. 4b). The H4K20me3 modification shifted toward the nuclear periphery during in vitro adipogenesis. On day 7 of adipogenic differentiation, H4K20me3 signals were preferentially localized in the peripheral part of the nuclei and were less abundant in the nuclear interior and the intermediate area than in undifferentiated cells (*P* < 0.0001). The signals of H4K20me3 modification also formed distinctive foci, which were arranged in a characteristic rim around the nuclear periphery on day 7 (Fig. 4a). The distributions of all heterochromatin marks measured as fluorescence intensity across nucleus are presented in Supplementary Fig. S8.

### Spatial distribution of euchromatin histone marks in MSC and adipocytes

**Figure 5:**
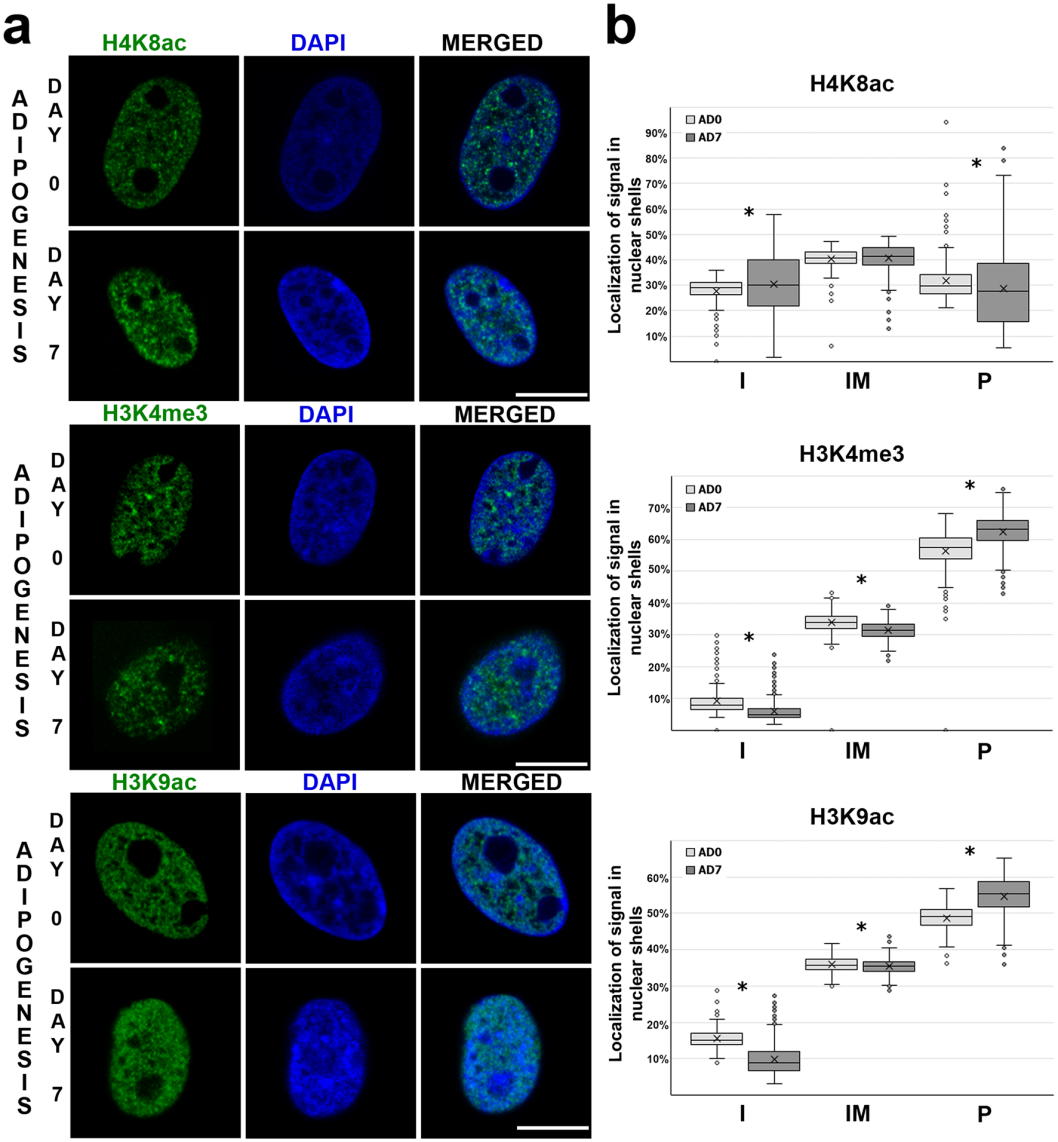
Spatial distribution of selected histone marks associated with euchromatin. (**a**) Distribution of H4K8ac, H3K4me3, and H3K9ac in a 3D nuclear space on days 0 and 7 of in vitro adipogenesis. The histone marks were visualized by indirect immunofluorescence (green). Nuclei were counterstained with DAPI (blue). Scale bar: 10 µm. (**b**) The graphs show the spatial distribution of immunofluorescent signals in 3D nuclear space. Statistically significant differences (*P* < 0.05) are marked with asterisks. Number of nuclei analyzed: H4K8ac: n = 551, H3K4me3: n = 594, H3K9ac: n = 653. Statistical data are shown in Supplementary Table 2.

The spatial distribution of selected euchromatin marks was analyzed, as described above, using shell analysis (Supplementary Fig. S7). In contrast to heterochromatin histone marks for euchromatin histone modifications, the signals were more dispersed throughout the nuclear volume (Fig. 5a, Supplementary Fig. S9). We observed that H4K8ac modification was evenly distributed in nuclei of undifferentiated cells (day 0) and adipocytes (day 7). When we compared days 0 and 7 of in vitro adipogenesis, the proportion of signals localized in nuclear interior increased (*P* = 0.0012), whereas on the nuclear periphery, the proportion of H4K8ac signals decreased (Fig. 5b) (*P* = 0.0044). Analysis of distribution of H3K4me3 histone mark showed that signals specific to this histone modification were localized mostly on the nuclear periphery. Moreover, when we compared undifferentiated cells (day 0) and adipocytes (day 7), we observed that it relocated towards the nuclear periphery (*P* < 0.0001) (Fig. 5b). Similar results were obtained for H3K9ac modification. The spatial distribution of H3K9ac mark showed that it was more present on the nuclear periphery than in the nuclear interior. With the progression of adipogenic differentiation, we observed that the percentage of signals located in the peripheral part of the nuclei increased (*P* < 0.0001) (Fig. 5b).

### Spatial nuclear distribution of repetitive sequences in MSC and adipocytes

**Figure 6:**
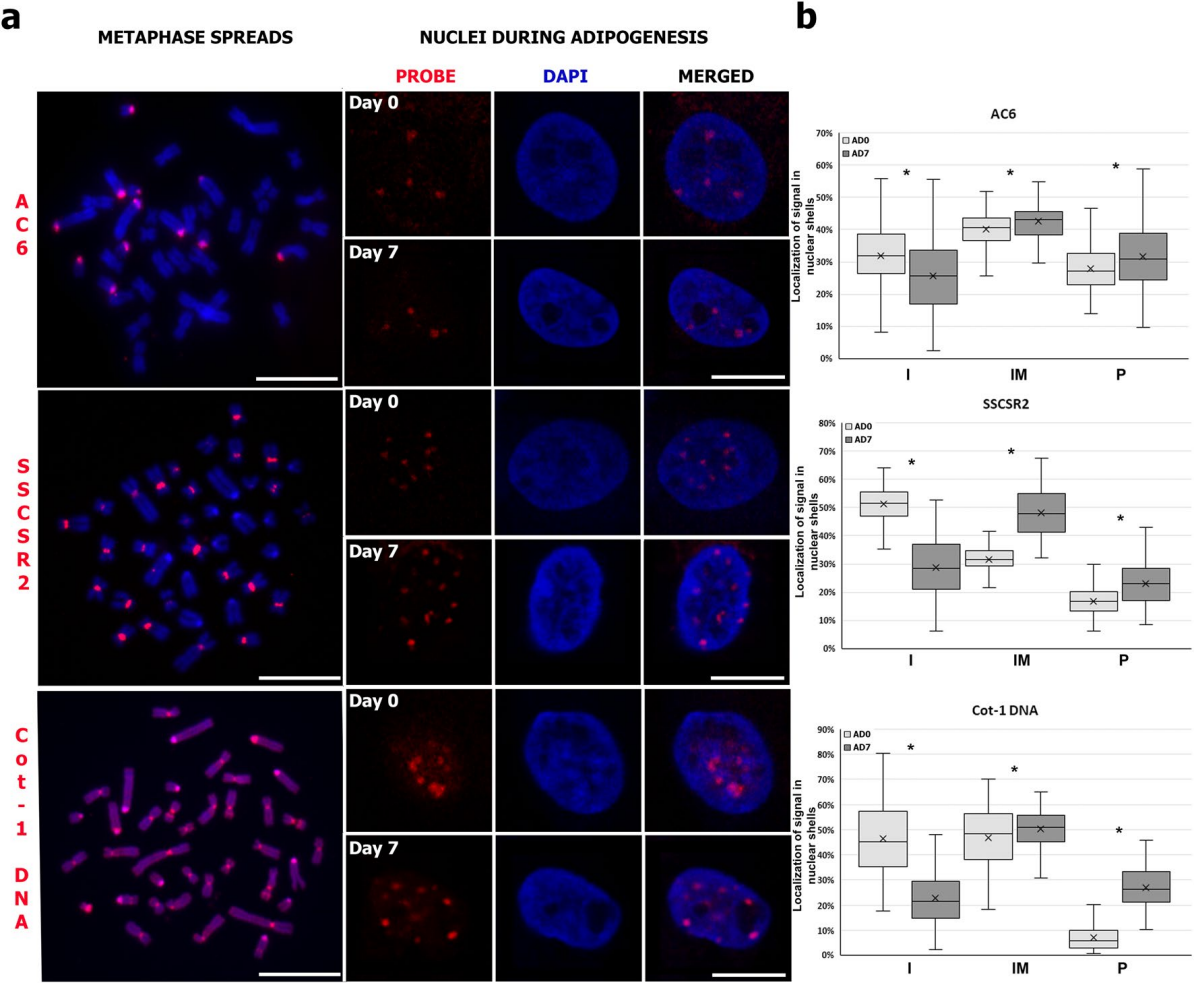
Spatial distribution of repetitive sequences in interphase nuclei during in vitro adipogenesis. (**a**) Representative images of 2D-FISH (metaphase chromosomes) and 3D-FISH (interphase nuclei) with probes specific to the centromeres of acrocentric chromosomes (AC6), the centromeres of bi-armed chromosomes (SSCSR2), and Cot-1 DNA. Probes were labeled with Cy3 (red) and chromatin was counterstained with DAPI (blue). Scale bar: 10 µm. (**b**) The graphs show the spatial distribution of hybridization signals in 3D nuclear space. Statistically significant differences (*P* < 0.05) are marked with asterisks. The number of nuclei analyzed was n = 256 for AC6, n = 256 for SSCSR2, and n = 251 for Cot-1 DNA.

In order to investigate whether the decrease in lamin A/C affects the nuclear localization of repetitive sequences during adipocyte differentiation, three probes were selected: a probe specific to the centromeric region of acrocentric porcine chromosomes (AC6); one specific to the centromeric region of biarmed porcine chromosomes (SSCSR2); and cot-1 DNA, enriched for repetitive DNA sequences. All probes were tested using 2-D FISH on metaphase chromosomes (Fig. 6a). Analysis of the spatial distribution of these sequences was performed using 3D-DNA FISH and confocal microscopy images with shell analysis (Supplementary Fig. S7). All the sequences shifted towards the nuclear periphery during adipogenesis (Fig. 6a,b). The centromeres of acrocentric chromosomes recognized by the AC6 probe mostly occupied the intermediate area of the nuclei. When day 0 of differentiation was compared with day 7, the proportion of signals localized in the nuclear interior was found to have decreased (*P* < 0.0001), whereas the proportion of AC6 signals was seen to have increased in the intermediate (*P* = 0.0004) and peripheral (*P* = 0.0013) regions (Fig. 6b). Analysis of the distribution of centromeric regions of the biarmed chromosomes detected by the SSCSR2 probe showed that FISH signals were localized mostly on the nuclear interior on day 0, but by day 7 these sequences were localized mostly in the intermediate region. When undifferentiated cells (day 0) were compared with adipocytes (day 7), sequences recognized by the SSCSR2 probe were found to have relocated towards the intermediate (*P* < 0.0001) and peripheral (*P* < 0.0001) parts of nuclei (Fig. 6b). Signals recognized by Cot-1 DNA probe were located mostly in the intermediate region. Comparing day 0 with day 7 of adipogenic differentiation showed a decrease in the proportion of Cot-1 DNA signals localized in the nuclear interior (*P* < 0.0001), coupled with an increase in the proportion of signals on the nuclear periphery (*P* < 0.0001) (Fig. 6b). Moreover, centromere clustering was observed in both undifferentiated cells and adipocytes (Supplementary Fig. S10, Supplementary Table 3). The number of clusters formed by centromeres of acrocentric chromosomes recognized by AC6 probe remained stable during adipocyte differentiation and formed 9 to 12 clusters per nucleus (representing 93% of the analyzed nuclei on day 0, and 92% on day 7). The centromeres of biarmed chromosomes, labeled with the SSCSR2 probe, formed from 10 to 16 clusters per nucleus in the undifferentiated cells (representing 80% of nuclei), while in adipocytes we observed a lower degree of clustering, with 19–22 signals per nucleus (representing 75% of nuclei). The repetitive DNA sequences recognized by Cot-1 DNA formed 6 to 11 clusters in the undifferentiated cells, whereas at day 7 of adipogenic differentiation we saw great variance in number of clusters, which ranged from 18 to 34 (representing 85% of nuclei).

## Discussion

It has been well established that different histone modifications are associated with various chromatin states^32^. In the present study, we characterized changes in the abundance and nuclear distribution of selected histone modifications and the three-dimensional nuclear organization of repetitive sequences during adipogenic differentiation. A previous study of global levels of eight histone modifications showed that they are stable during adipogenesis in 3T3-L1 and 10T1/2 cell lines^33^. The authors of that study observed only dynamic histone modification patterns in specific genes. In our study, we detected an increase in two euchromatin marks, H4K8ac and H3K9ac. Increased levels of H3K9ac, H3K4me2, and H4K8ac were also seen during differentiation of human MSCs treated with abexinostat, a chemical compound that promotes adipogenesis^34^; since it inhibits histone deacetylases, this indicates that correct histone acetylation is crucial for adipocyte formation. It has previously been shown that histone acetylation increases at the promoters of adipogenic genes, while a decrease in the expression of histone deacetylases takes place throughout the differentiation process^35^.

We also found that the heterochromatin marks H3K27me3 and H4K20me3 significantly increased during adipogenic differentiation. H3K27me3 modification is a polycomb heterochromatin marker that is generally associated with gene repression^36^. It has been observed that global levels of H3K27me3 increase in many lineages as cells differentiate^37^. Studies of histone H3K27 methyltransferase EZH2 and demethylase KDM6A have revealed their regulatory role during human adipogenic differentiation, where they act as positive and negative regulators, respectively^38^. The significant increase in H3K27me3 levels observed in this study indicates the importance of this histone modification during adipocyte formation. H4K20me3 modification is a hallmark of pericentromeric heterochromatin, but it is also observed near nucleoli and at the nuclear periphery^39^. Global increases in H4K20me3 modification have also been reported during other differentiation processes, such as those of skeletal muscle, epidermal cells, and neuronal cells^40–42^. H4K20me3 also increases in quiescent cells^43^, while it decreases in embryonic stem (ES) cells^44^. It seems that this modification plays a role in terminal differentiation of cells. Adipocytes, as fully developed nondividing cells, are an example of this. We did not observe here any changes in H3K9me3 level. Increases in H4K20me3 and decreases in H3K9me3 were seen in fibroblasts derived from patients with laminopathies (Hutchinson-Gilford progeria syndrome)^45^. This upregulation of H4K20me3 was linked with the blocking of telomere elongation, since this modification also marks telomeric heterochromatin^46^.

H4K20me3 and H3K9me3 are two classic markers of constitutive heterochromatin that act together with heterochromatin protein 1 (HP1) to establish pericentromeric heterochromatin^47,48^. In an interphase nucleus, this pericentric constitutive heterochromatin may form roundish bodies called chromocenters. Each chromocenter consists of multiple pericentric regions of different chromosomes. Reorganization of chromocenters may occur during development and differentiation processes^49^. We previously showed that the chromocenters identified by the anti-HP-1α antibody are reorganized during porcine adipogenesis, from clearly visible foci in MSCs at early stages of differentiation, to more diffuse in the terminal differentiation^4^. Here, after immunostaining with anti-H3K9me3 and anti-H4K20me3 antibodies, strong signals were observed in chromocenters and in nucleoli in MSCs. A similar distribution of H3K9me3 marks was reported in fibroblasts, where it was found near nucleoli and at nuclear periphery; the abundance of this histone mark decreased with cell senescence^50^. Redistributions in the 3D nuclear space of these histone marks were detected during adipogenesis. We found that, in mature adipocytes, H4K20me3 was highly abundant at the nuclear periphery and forms a rim around the nuclear envelope. A similar pattern has also been observed for H3K9me3, but with weaker signals. It seems that enrichment of heterochromatin at the nuclear periphery in mature adipocytes may play a role in the maintenance of nuclear morphology. We also found that repetitive sequences specific to α-satellite sequences underwent relocalization toward the nuclear periphery in mature adipocytes. We noted aggregation of centromeric sequences and formation chromocenters, which is a characteristic feature also observed in other porcine cell types such as lymphocytes, neutrophils, and sperm^51,52^. Interactions between heterochromatic regions have been identified as responsible for the segregation of chromatin and the compartmentalization of nuclei^16^. In conventional nuclei, the association of heterochromatin and lamina is also needed. We have shown that although low level of lamins are observed in adipocytes, heterochromatin can nonetheless be tethered to the nuclear periphery, allowing the conventional nuclear architecture to be established.

The expression of lamin A/C is dependent on cell type and stage of development or differentiation. During development, only LBR is expressed, but is replaced by lamin A/C during differentiation. However, it was found that lamin A/C is also present in early porcine embryos (from 1-cell to 8-cell stage) but is lacking from later embryonic stages^53^. The expression of both these proteins in differentiated cells is not very common^15^. Here, we shown that the expression of both nuclear envelope proteins significantly decrease during adipogenesis. Although the decrease in lamin A/C during adipogenesis has been described^4,7^, there is not much information on LBR. Only Buxboim et al.^19^ have reported an increase in LBR in adipocytes, contrary to our results. Low expression levels of lamin A/C and LBR are typical of rod cells, where inverted chromatin has been found^15^. Moreover, knocking lamin A/C and LBR out in other cell types also leads to the same inverted patterns, which indicates the role of these proteins in maintaining peripheral heterochromatin^15^. During differentiation of other cell types, such as neutrophils, increased expression of LBR and decreased expression of lamin A/C have been observed^54^. Sufficient levels of LBR are required during differentiation of granulocytes to achieve the unusual lobulated nuclear shape^55^. Lukášová et al.^56^ found that the loss of lamin B receptor is necessary for cells to transition to senescence, and observed that centromeric heterochromatin relocates by shRNA from the inner nuclear membrane to the nucleoplasm, not only in senescent cells, but also in cells with reduced expression of LBR. Lamin A/C and LBR have been implicated in chromatin organization as well as in gene positioning. LBR plays a role in the aggregation of inactive olfactory receptor (OR) gene clusters in olfactory sensory neurons^57^. The repositioning of genes from the nuclear lamina during many differentiation processes is a well-known mechanism of transcriptional activation^58,59^. Our previous experiments have shown the relocation of adipogenic genes from the nuclear periphery during differentiation^6,8,23^, but further studies are needed to elucidate if the decrease in lamin plays a direct role in this reorganization.

It was previously thought that lamins dictate nuclear morphology^60–62^. The aberrant nuclear morphology seen in many diseases, such as cancers and laminopathies, has been linked to alterations of lamins^63,64^. Surprisingly, it was recently shown by Stephens et al.^65^ that the key factors underlying nuclear mechanics and morphology are chromatin histone modifications. These authors found that treating mammalian cells with histone deacetylase inhibitors, to increase euchromatin or histone methyltransferase inhibitors and decrease heterochromatin, results in a softer nucleus and nuclear blebbing without alternations in the amount or organization of lamins. Furthermore, increasing the amount of heterochromatin rescued the nuclear morphology in Hutchinson–Gilford progeria syndrome patient cells. These findings are in the line with our observation that increases in heterochromatin histone marks at the nuclear periphery may allow the maintenance of nuclear morphology in fully developed adipocytes. Studies of interactions between chromatin state and nuclear mechanics and morphology are important to better understand the regulation of adipocyte differentiation. Much evidence has been presented recently of how mechanotransduction affects nuclear spatial organization and gene expression in stem cells^66^. One study on the differentiation of human MSCs into adipocytes has shown that changes in nuclear morphology are a prerequisite for adipocyte maturation, and that cells with elongated nuclear shapes are those that did not efficiently undergo adipogenesis^67^.

In conclusion, we have shown that there is decreased expression of two nuclear envelope proteins, lamin A/C and LBR, during porcine adipogenesis. Although these proteins have been implicated in maintenance of heterochromatin in peripheral nuclear location, we found that repetitive sequences underwent relocalization toward the nuclear periphery in mature adipocytes. The heterochromatin histone marks, H4K20me3 and H3K27me3, increased during adipogenesis, and were found to be enriched in the nuclear periphery in fully developed adipocytes. Such reorganization may allow the nuclear morphology to be maintained in cells with increased lipid production and decreased levels of lamins. We also observed increased levels of H3K9ac and H4K8ac modifications and their redistribution in nuclear space, which point to the role of histone acetylation in adipocyte formation.

## Materials and methods

### Ethics statement

All procedures were performed in accordance with the “Act on the protection of animals used for scientific purpose” of the Republic of Poland, which complies with the European Union Legislation for the protection of animals used for scientific purposes. All animal procedures were approved by the Local Ethical Commission on Experiments on Animals at the Poznan University of Life Sciences, Poznan, Poland (approval No. 57/2012). All methods were performed in accordance with the relevant guidelines and regulations.

### Cell culture

Mesenchymal stem cells were isolated from adipose tissue (AD-MSC) of a three-month-old Large White pig, as described earlier^68^. Cells were cultured in Advanced DMEM supplemented with 10% FBS, 5 ng/ml FGF-2 (PromoKine), 2 mM l-Glutamine (PAA), 1 mM 2-mercaptoethanol (Sigma), 1 × antibiotic antimycotic solution (Sigma) and 1 × MEM NEAA (Thermo Fisher) at 37 °C in 5% CO_2_. Adipogenesis was induced when the cells reached confluency by culturing cells in adipogenic medium, which consisted of Advanced DMEM (Gibco), 10% FBS (Sigma), 1 × antibiotic antimycotic solution (Sigma), 1 × MEM NEAA (Thermo Fisher), 5 ng/ml FGF-2 (PromoKine), 1 × Linoleic Acid Albumin , 1 × ITS, 1 µm Dexamethasone (Sigma), 100 µm Indomethacin (Sigma) and 50 mM IBMX (Sigma). The adipogenic differentiation lasted for seven days and was monitored under a phase-contrast microscope (TS100 Eclipse, Nikon).

### Monitoring of lipid droplet formation

The accumulation of lipid droplets was confirmed by BODIPY staining. Cells were fixed with 4% paraformaldehyde in PBS (w/v) for 10 min at room temperature and washed with PBS three times. The cells were then incubated with BODIPY (Life Technologies) in PBS (2.7 µg/ml) and washed three times in PBS. The nuclei were counterstained with DAPI in Vectashield medium (Vector Laboratories).

### RNA isolation and real-time PCR

Total RNA was extracted from cells on each day of the seven-day adipogenesis process, beginning with the undifferentiated cells on day 0. TriPure Isolation Reagent (Roche Diagnostics) was employed following the manufacturer’s instructions. The concentrations and purity of RNA samples were determined by a NanoDrop spectrophotometer (Thermo Scientific). The cDNA was reversely transcribed from 2 µg of total RNA samples. The *LMNA* and *LBR* transcript levels were assessed using a LightCycler 480 SYBR Green I Master kit (Roche Diagnostic) with a LightCycler 480 II (Roche Life Science). All samples were analyzed in triplicate. Standard curves were designed as tenfold dilutions of the PCR products. Relative transcript levels were quantified using the 2^−ΔΔCT^ method^69^ with undifferentiated cells (day 0) used as a calibrator. The relative transcript quantification was also calculated in relation to the reference gene. The *RPL27* gene (ribosomal protein L27) was used as a reference. The sequences of primer sets were: *LMNA* F: 5′-CTCAAGGCACGCAATACCAAG-3′, R: 5′-TGTTTCTTGGCCTCACCCAG-3′; *LBR* F: 5′-GGATTGATTGGCTGGGTGGT-3′, R: 5′-CACACTAGATCCCCGAAGGC-3′; *RPL27* F: 5′-GCAAAGCGGTCATCGTAAA-3′, R: 5′-GCAAAGCGGTCATCGTAAA-3’.

### Selection of specific histone modifications

Histone modifications were selected because they accumulate specifically on transcriptionally active and repressed chromatin, and have previously been used in nuclear architecture studies for the recognition of the main classes of chromatin^14,16^. The histone modifications we investigated were H3K9me3 (constitutive heterochromatin marker), H3K27me3 (facultative heterochromatin marker), H4K20me3 (constitutive heterochromatin marker), H4K8ac (euchromatin marker), H3K4me3 (euchromatin marker), and H3K9ac (euchromatin marker).

### Immunofluorescent staining

Indirect immunofluorescent staining was performed on cells grown on glass coverslips on day 0 (undifferentiated) and day 7 (adipocytes). Cells were fixed with 4% PFA in PBS (w/v) and washed with PBS. Permeabilization was performed for 15 min at RT in 0.5% Triton X-100 in PBS. The cells were then blocked in 3% BSA in PBS for 30 min at RT, for visualization of lamin B receptor blocking was performed in 1% BSA in PBS for 20 min. Primary antibodies were diluted in 1.5% BSA in PBS at different proportions: anti-lamin A/C produced in mouse (Sigma-Aldrich) was diluted at 1:100, anti-lamin B receptor antibody (Abcam) was diluted at 1:50, anti-histone H3 (tri methyl K9) antibody (Abcam) was diluted at 1:80, anti-histone H3 (tri methyl K27) antibody (Abcam) at 1:100, anti-histone H4 (tri methyl K20) antibody (Abcam) at 1:100, anti-histone H4 (acetyl K8) antibody (Abcam) was diluted at 1:100, anti-histone H3 (tri methyl K4) antibody (Abcam) at 1:20, and anti-histone H3 (acetyl K9) antibody (Abcam) was diluted at 1:70. Incubation with primary antibodies was performed overnight at 4 °C and the cells were then washed three times in PBS. After washing, the cells were incubated with secondary antibodies labeled with TRITC anti-mouse IgG (whole molecule)-TRITC antibody produced in rabbit (Sigma) or Alexa Fluore 488 (Goat polyclonal anti-rabbit IgG with Alexa Fluor 488) diluted at 1:200 in 1.5% BSA in PBS. Cells were then washed three times in PBS. For the immunofluorescent protein labeling of lamin A/C, additional lipid droplets were stained with BODIPY (Life Technologies). Cells were incubated with BODIPY at a concentration of 2.7 µg/ml in PBS, and then washed three times in PBS. The nuclei were counterstained in DAPI mounted in Vectashield medium (Vector Laboratories).

### Protein isolation

Histone proteins were isolated using Histone Extraction Kit (Abcam), following the manufacturer’s instructions on days 0 and 7 of adipogenesis in three biological replications. Briefly, cells were detached from plates by trypsinization in 0.25% Tripsin-EDTA solution (Sigma) for 5 min at 37 °C, in line with the standard procedure. Cells were then resuspended in 1 × Pre-Lysis Buffer at a concentration of 10^7^ cells/ml and lysed for 10 min with gentle stirring at 4 °C. The cells were then collected by centrifugation at 10,000*g* for 1 min at 4 °C. Cells were resuspended in Lysis Buffer (200 µl per initial 10^7^ cells) and incubated at 4 °C for 30 min. The cell suspension was then centrifuged at 12,000*g* for 5 min at 4 °C, and the supernatant, containing acid-soluble proteins, was transferred to a new vial. Finally, 3 µl the Balance-DTT Buffer was added for each 10 µl of supernatant. Protein concentration was measured using Qubit 2.0 Fluorometer (Invitrogen) with Protein assay kit (Invitrogen).

### Western blot analysis

Protein samples were mixed with Laemmli buffer (BioRad) and β-mercaptoethanol (Sigma). Each sample was analyzed in triplicate. Next, samples were denatured, loaded on a gel (0.1 µg protein per sample), and separated by electrophoresis (60 min, 125 V). Then proteins were blotted onto a polyvinylidene difluoride (PVDF) membrane using a Trans-Blot Turbo Transfer System (BioRad). Membranes were blocked with 3% BSA for 1 h at RT and incubated with primary antibodies (anti-histone H3 (tri methyl K9) antibody (Abcam), anti-histone H4 (tri methyl K20) antibody (Abcam), anti-histone H4 (acetyl K8) antibody (Abcam), anti-histone H3 (tri methyl K4) antibody (Abcam), anti-histone H3 (tri methyl K27) antibody (Abcam), and anti-histone H3 (acetyl K9) antibody (Abcam)) in TBST for 16 h at 4 °C. The membranes were then washed three times for 10 min with TBST, and were incubated with secondary antibody (anti-rabbit) for 1 h at RT and washed. The chemiluminescence reaction was performed with Clarity Western ECL Substrate (BioRad). Signals were visualized with ChemiDoc Imaging System (BioRad), and signal intensities were measured using ImageLab software (BioRad).

### Hybridization probes

The Cot1-DNA probe was obtained by labeling of Porcine Hyblock DNA (Applied Genetics Laboratories) using the random-priming method with biotin-16-dUTP (Roche Diagnostic) and employing a BioPrimer Array CGH genomic labeling system (Thermo Fisher Scientific). The SSCSR2 and AC6 probes were generated by PCR amplification with following primers: SSCSR2A-F: 5′-agcgcttgcctagttctcacctagc-3′; SSCSR2B-R: 5′-atcctgagccaagcggcattgg-3′; AC6-F: 5′-attccatgcagcagcgtgattga-3′; AC6-R: 5′-tcaatcacgctgcatggaat-3′^70,71^. These probes were labeled with biotin-16-dUTP (Roche Diagnostic). The Cot-1 DNA probe enriched in repetitive DNA sequences labels the centromeres of all porcine chromosomes (38 signals on metaphase spread); probe AC6 recognizes the consensus 14-bp motif, which is present in all acrocentric chromosomes (12 FISH signals on metaphase spread); and probe SSCSR2 labels all biarmed chromosomes except for chromosomes no. 10 and 12 (22 FISH signals on metaphase spread). Probes specificity was checked on porcine metaphase chromosomes using 2D-FISH. Slides were analyzed under a Nikon E600 Eclipse fluorescence microscope.

### 3D fluorescence in situ hybridization (3D-FISH)

3D-FISH was performed following the method described by Szczerbal et al.^6^. Mesenchymal stem cells were grown and differentiated into adipocytes directly on the microscope slides. Cells were fixed in 4% paraformaldehyde in PBS (w/v) for 10 min at days 0 and day 7 of differentiation. Cells were then incubated in 20% glycerol in PBS (v/v) and exposed to seven repeated freeze–thaw cycles in liquid nitrogen. The cells were then treated with 0.1 M HCl for 5 min and equilibrated in 50% formamide in 2 × SSC. The probes were denatured in 75 °C for 10 min. The nuclei were denatured at 75 °C in 70% formamide in 2 × SSC for 3 min and in 50% formamide in 2 × SSC for 1 min. The slides were placed in a humid chamber at 37 °C for two days for hybridization. The slides were then washed three times in 50% formamide in 2 × SSC for 6 min and three times in 2 × SSC for 6 min at 42 °C. The slides were equilibrated in 4 × SSC/0.05% Tween-20, and then blocked with 3% BSA in 4 × SSC/0.05% Tween-20 for 30 min at RT. The probes were detected by incubation for 60 min with Cy3-Streptavidin (GE Healthcare) diluted at 1:200 in with 3% BSA in 4 × SSC/0.05% Tween-20 at RT. The slides were washed three times in 4 × SSC/0.05% for 6 min at 42 °C and for 5 min in PBS at RT. The nuclei were counterstained with DAPI in Vectashield mounting medium (Vector Laboratories).

### Confocal microscopy and image analysis

Images were acquired by Carl Zeiss LSM 880 confocal microscope with Airyscan using a 63 ×/1.4 NA Plan-Apochromat oil objective. Lasers with three excitation lines were used: 560 nm for TRITC or Cy3, 488 nm for FITC or BODIPY, and 420 nm for DAPI. The pinhole, filters, and objectives were kept at constant settings throughout the examination of all slides. Each Z-stack consisted of 30–50 images with a step size of 0.25 µm and a pixel size of 71 nm × 71 nm × 250 nm. The image stacks were processed by Airyscan to increase the signal-to-noise ratio and image resolution. To analyze lamin A/C abundance, the signal intensities of individual nuclei were measured in Fiji with the Measure function from Z-Projections, obtained as a sum of stacked images. For analysis of spatial distribution Z-stacks were analyzed in Tango^72^, the nuclei were segmented using its Simple Segmenter function, and signals from histone marks or hybridization signals from 3D FISH were segmented with the Hysteresis Segmenter. The spatial distribution of the histone marks was examined with the Shell Analysis function, which shows the proportion of signals localized in each of the three concentric shells of equal volume in the three-dimensional nuclear space (Supplementary Fig. S5). To determine the abundance of lamin A/C, lamin B receptor, and selected histone modifications the signal intensities for individual nuclei were measured with the Signal Quantification function in Tango^72^ and the results normalized by fluorescence intensities for DAPI.

### Statistical analysis

The statistical analysis was performed using SAS 9.4 software. All variables were tested for normal distribution with the Kolmogorov–Smirnov test. The differences in lamin A/C fluorescence intensity were analyzed with Student’s *t*-test. The transcript levels of the *LMNA* gene were compared using ANOVA and the post-hoc Duncan test. The transcript levels of *LBR* gene were analyzed with Kruskal–Wallis test and the post-hoc Dunn test. The signal intensities obtained from western blot analysis were compared with Student’s *t*-test. Depending on the data distribution, the positions of histone marks and FISH signals in 3D nuclear space were analyzed using ANOVA or the Kruskal–Wallis test and the post-hoc Tukey test or the post-hoc Dunn test.

## Supplementary Information

Supplementary Information.

## Data Availability

The datasets during and/or analyzed during the current study are available from the corresponding authors on reasonable request.
