# Supplementary material for: Alteration of active and repressive histone marks during adipogenic differentiation of porcine mesenchymal stem cells
Source: Sci Rep. 2021 Jan 14;11:1325. doi: 10.1038/s41598-020-79384-x (PMC7809488; doi:10.1038/s41598-020-79384-x)
Supplement: Supplementary file 1 — Supplementary Information. [file 41598_2020_79384_MOESM1_ESM.docx]

**Supplementary Information**

**Alteration of active and repressive histone marks during adipogenic differentiation of porcine mesenchymal stem cells**

**Joanna Stachecka,^1^ Pawel A. Kolodziejski,^2^ Magdalena Noak,^1^ Izabela Szczerbal^1*^**

*^1^Department of Genetics and Animal Breeding, Poznan University of Life Sciences, Wolynska 33, 60-637 Poznan, Poland*

*^2^Department of Animal Physiology, Biochemistry and Biostructure, Poznan University of Life Sciences, Wolynska 35, 60-637 Poznan, Poland*

*Corresponding author:

Prof. Izabela Szczerbal

email address: izabel@up.poznan.pl


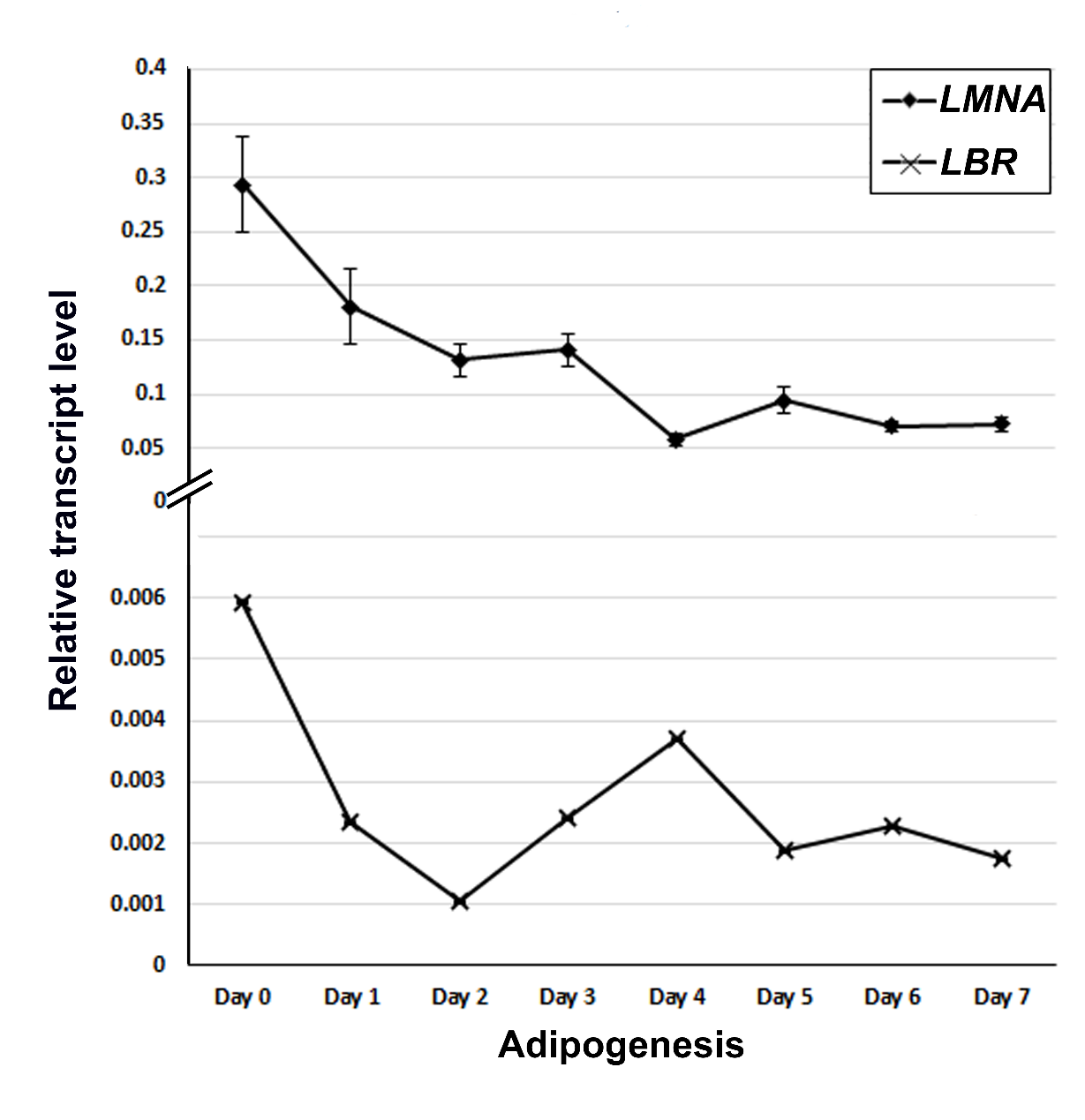


**Supplementary Figure S1.** Comparison of nuclear envelope proteins transcript levels during porcine *in vitro* adipogenesis. The *LMNA* and *LBR* transcript levels were normalized by a reference gene *RPL27*. Error bars show SD.

**
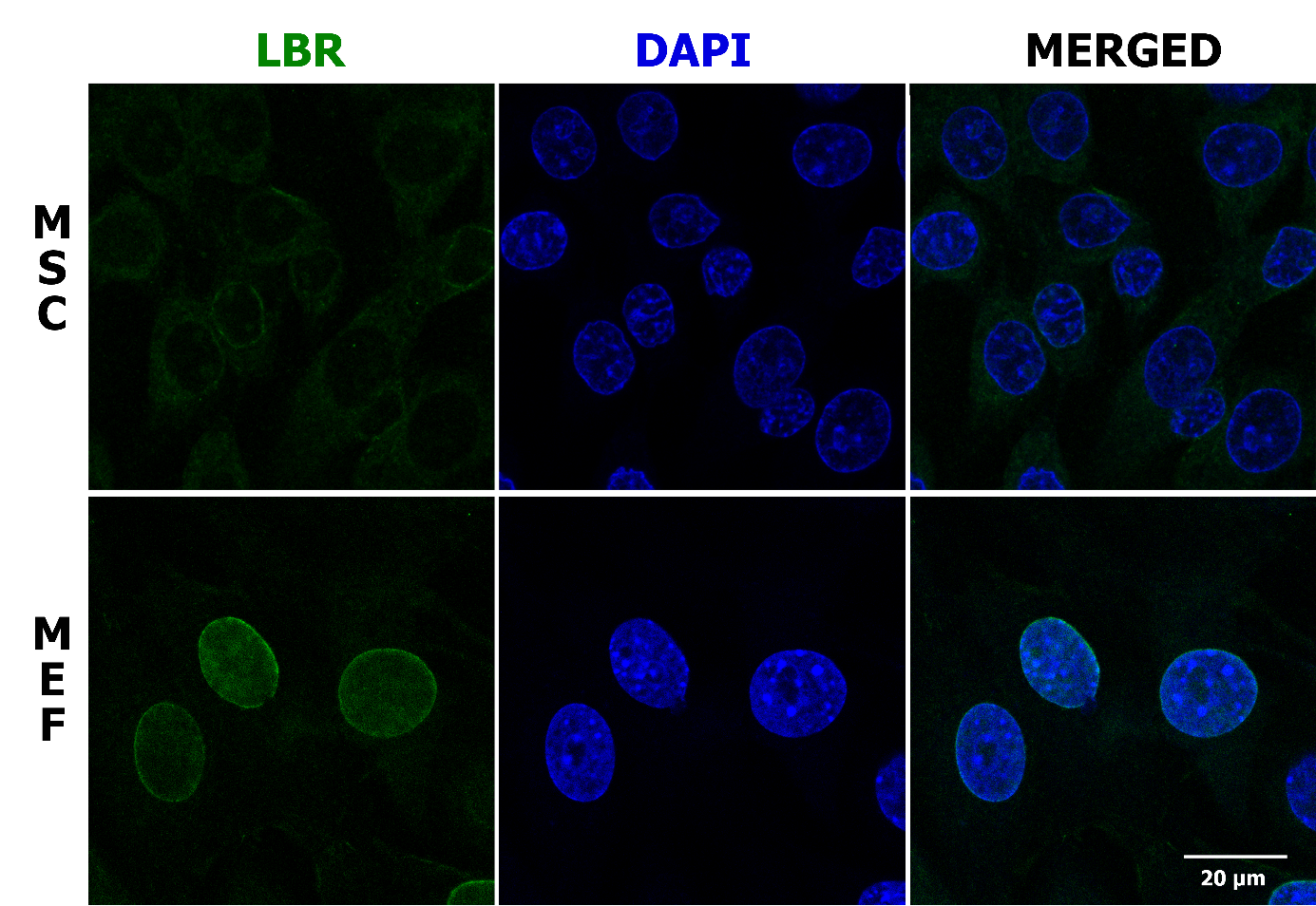
**

**Supplementary Figure S2**. Visualization of lamin B receptor (LBR) in porcine mesenchymal stem cells (MSC). Very weak positive signals for LBR were observed in undifferentiated cells. The specificity of the anti-lamin B receptor antibody staining was confirmed on mouse embryonic fibroblasts (MEF). The lamin B receptor was visualized by indirect immunofluorescence (green), nuclei were counterstained with DAPI (blue).


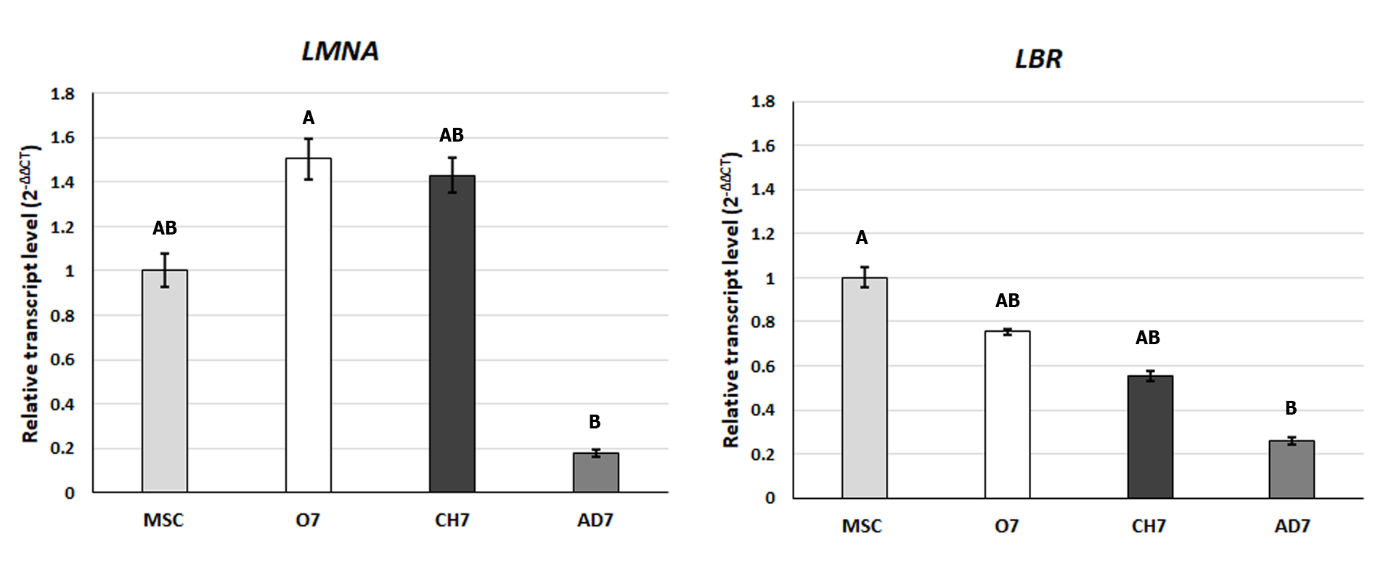


**Supplementary Figure S3.** Expression of lamin A/C and lamin B receptor during porcine *in vitro* osteogenesis, chondrogenesis, and adipogenesis. Osteogenic and chondrogenic differentiations were induced with the StemPro Osteogenesis Differentiation Kit (Gibco) and StemPro Chondrogenesis Differentiation Kit (Gibco), respectively. Differentiation was conducted for seven days (O7, CH7, AD7). The *PPIA* was used as a reference gene for osteogenesis and chondrogenesis (primer sequences available in Kociucka *et al.*, 2017, *J Anim Sci.* 95: 4514–4519). Relative transcript levels of the *LMNA* and *LBR* genes on day 7 of differentiation in comparison to undifferentiated MSC. Error bars show SDs. Different capital letters indicate significant differences between analyzed samples (P < 0.05).

| **H3K9me3**  **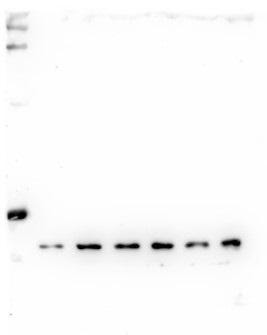** | **H3K9ac**  **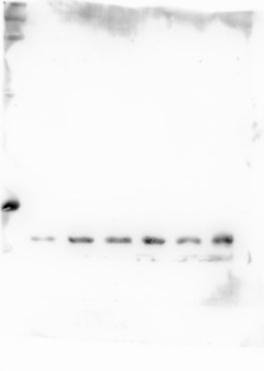** |
| --- | --- |
| **H3K4me3**  **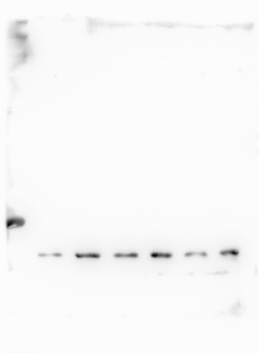** | **H3K27me3**  **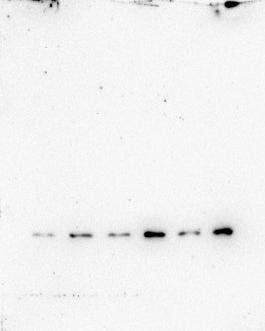** |
| **H3pan**  **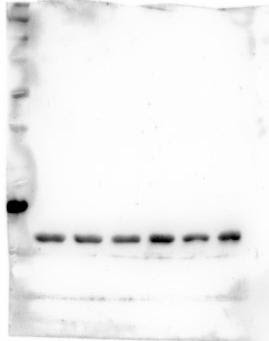** |  |

**Supplementary Figure S4.** Full length blots for histone H3. Molecular weight marker is shown in the far left lane. All blots are from the same membrane. Blot images are shown as chemiluminescence images.

| **H4K20me3**  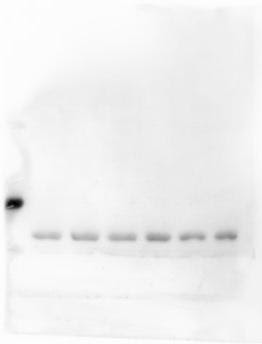 | **H4K8ac**  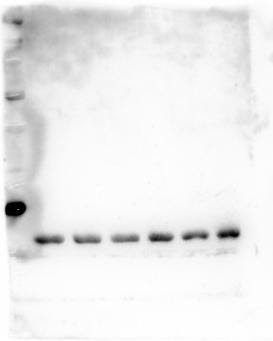 |
| --- | --- |
| **H4pan**  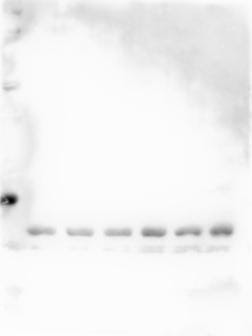 |  |

**Supplementary Figure S5.** Full length blots for histone H4. Molecular weight marker is shown in the far left lane. All blots are from the same membrane. Blot images are shown as chemiluminescence images.


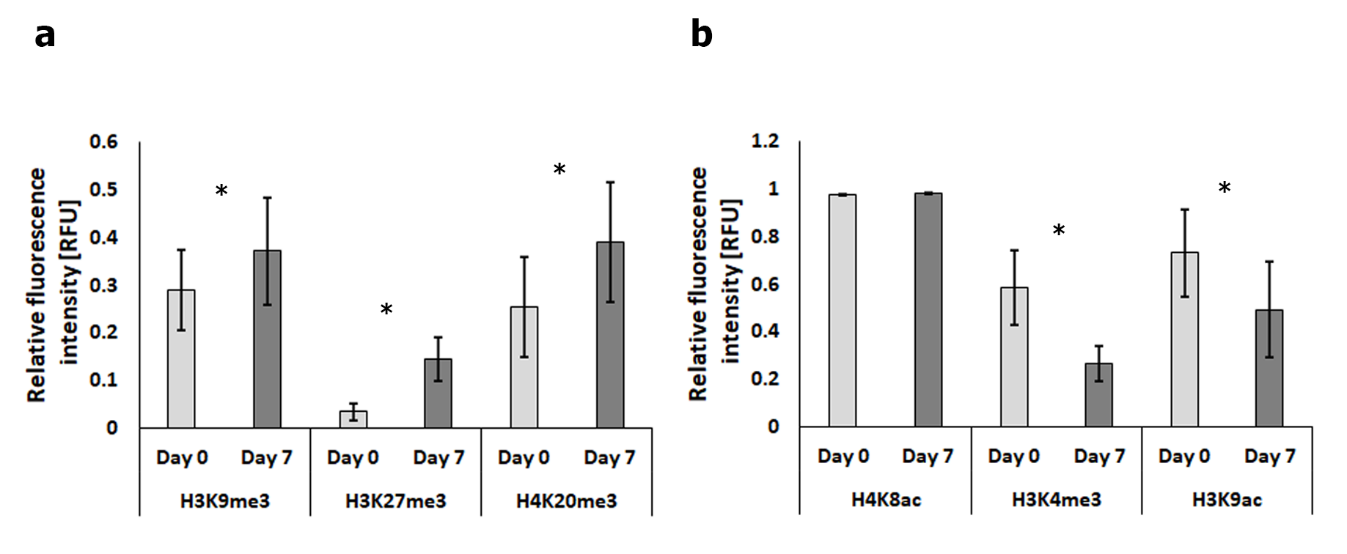


**Supplementary Figure S6.** Relative fluorescence intensity levels measured for selected histone marks. Measurements of heterochromatin (a) and euchromatin (b) histone mark signals were normalized by the DAPI fluorescence intensity. Error bars shows SD. Statistically significant differences (P < 0.05) are marked with asterisks. The number of nuclei analyzed was n = 497 for H3K9me3, n = 243 for H3K27me3, n = 604 for H4K20me3,  n = 551 for H4K8ac, n = 594 for H3K4me3, and n = 653 for H3K9ac.


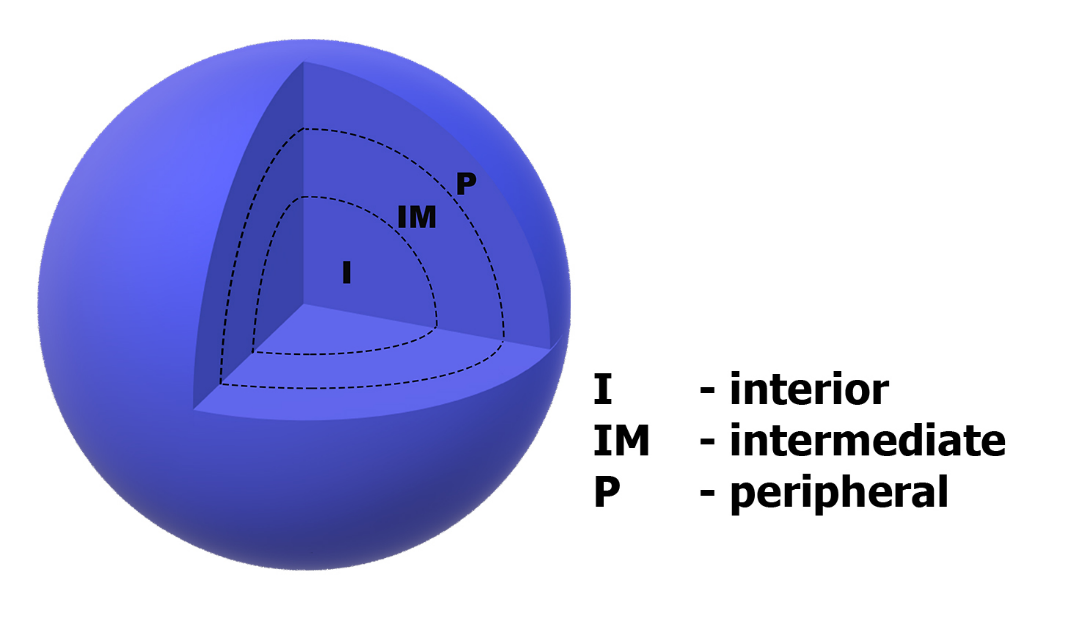


**Supplementary Figure S7**. Schematic representation of the nucleus. Three concentric shells of equal volume are distinguished within the 3D nuclear space: nuclear interior, intermediate shell, and peripheral shell. All analyzed signals in the three-dimensional nuclear space were divided according to their localization with Shell analysis function in Tango plug-in to FIJI software. The results show the percentage of signals localized in each of the nuclear shells.


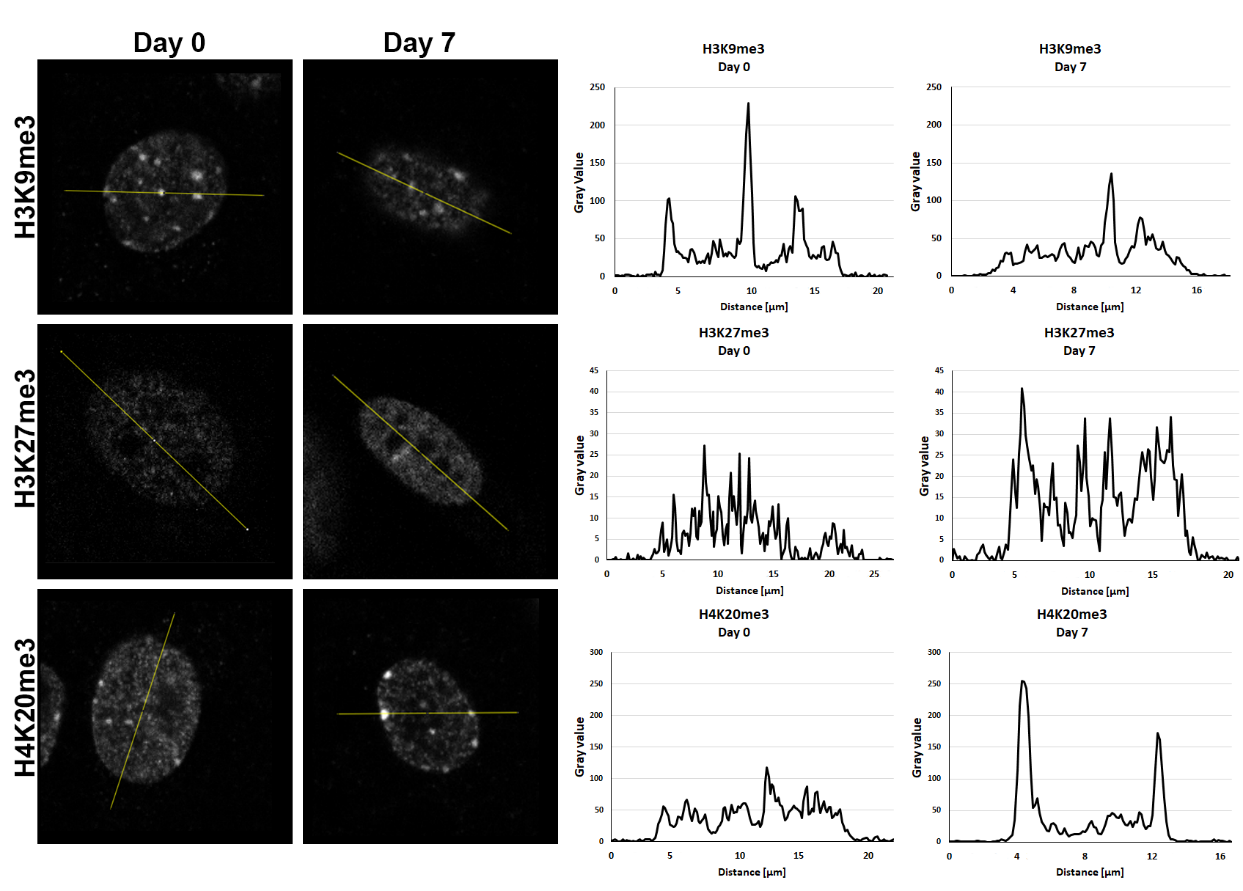


**Supplementary Figure S8.** Fluorescence intensity plot profiles of heterochromatin histone marks. Profiles of fluorescence intensity for H3K9me3, H3K27me3, and H4K20me3 histone marks were acquired with FIJI software.

**
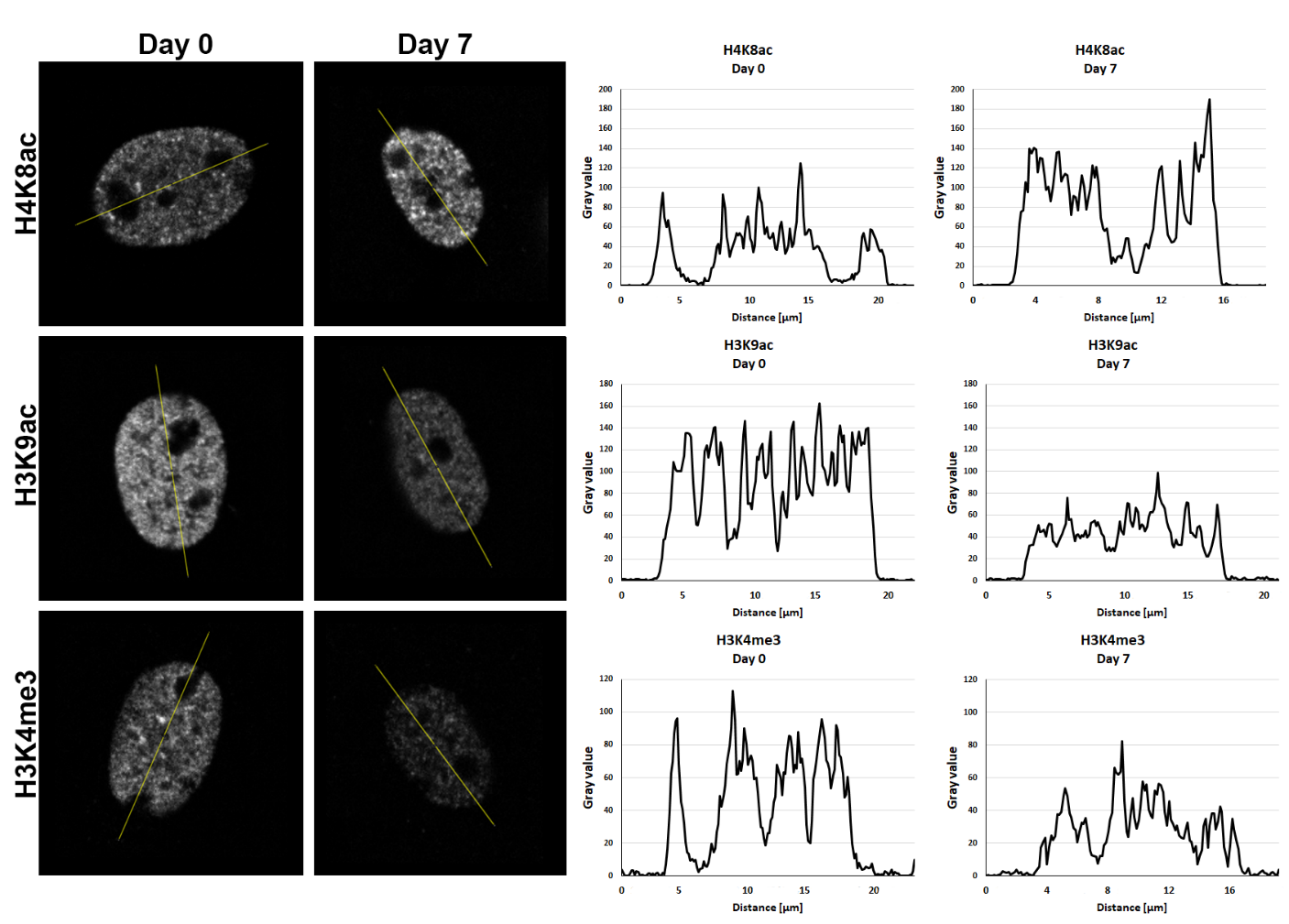
**

**Supplementary Figure S9.** Fluorescence intensity plot profiles of euchromatin histone marks. Profiles of fluorescence intensity for H4K8ac, H3K9ac and H3K4me3 histone marks were acquired with FIJI software.


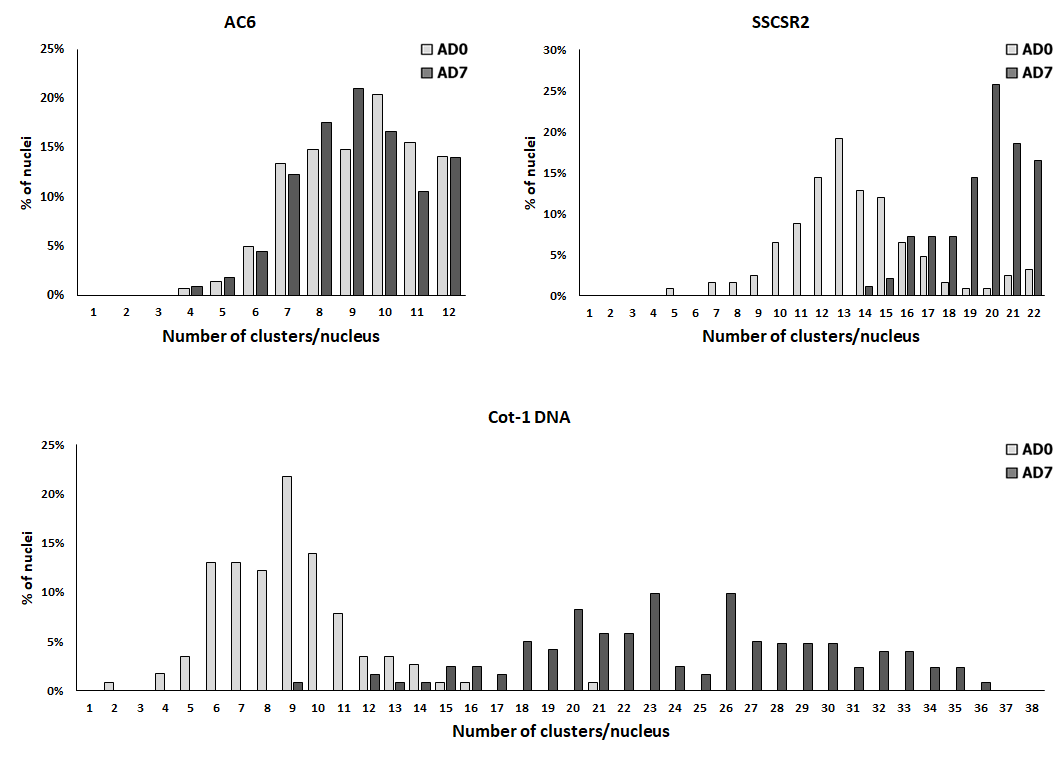


**Supplementary Figure S10.** Centromere clustering in undifferentiated cells (AD0) and adipocytes at day 7 of adipogenic differentiation (AD7). Percentage of nuclei with different number of FISH signals showing degree of centromere clustering analyzed with AC6, SSCSR2 and Cot-1 DNA probes.

**Supplementary Table 1.** Statistical data supporting results presented on Figure 4b. Data shows mean and variance calculated for percentage of signals specific for selected heterochromatin histone marks residing in interior (I), intermediate (IM), and peripheral (P) shells.

| **Heterochromatin histone marks** | | | | | |
| --- | --- | --- | --- | --- | --- |
|  | **Shell** | **Day** | **Mean** | **Variance** | **p-value** |
| **H3K9me3** | **I** | Day 0 | 21.7971 | 0.4509 | 0.0238 |
|  |  | Day 7 | 23.6951 | 0.9605 |  |
|  | **IM** | Day 0 | 36.5339 | 0.1563 | <.0001 |
|  |  | Day 7 | 40.7877 | 0.3693 |  |
|  | **P** | Day 0 | 41.6719 | 0.7696 | <.0001 |
|  |  | Day 7 | 35.5153 | 1.7268 |  |
| **H3K27me3** | **I** | Day 0 | 69.1569 | 0.2446 | <.0001 |
|  |  | Day 7 | 49.4181 | 0.1929 |  |
|  | **IM** | Day 0 | 25.1731 | 0.0734 | <.0001 |
|  |  | Day 7 | 37.5108 | 0.0819 |  |
|  | **P** | Day 0 | 05.6701 | 0.0963 | <.0001 |
|  |  | Day 7 | 13.0712 | 0.1433 |  |
| **H4K20me3** | **I** | Day 0 | 22.1137 | 1.2335 | <.0001 |
|  |  | Day 7 | 13.8352 | 0.3175 |  |
|  | **IM** | Day 0 | 41.1233 | 0.5723 | <.0001 |
|  |  | Day 7 | 36.6042 | 0.1187 |  |
|  | **P** | Day 0 | 36.7580 | 2.4956 | <.0001 |
|  |  | Day 7 | 49.5605 | 0.5936 |  |

**Supplementary Table 2.** Statistical data supporting results presented on Figure 5b. Data shows mean and variance calculated for percentage of signals specific for selected euchromatin histone marks residing in interior (I), intermediate (IM), and peripheral (P) shells.

| **Euchromatin histone marks** | | | | | |
| --- | --- | --- | --- | --- | --- |
|  | **Shell** | **Day** | **Mean** | **Variance** | **p-value** |
| **H4K8ac** | **I** | Day 0 | 27.8582 | 0.27667 | 0.0012 |
|  |  | Day 7 | 30.4478 | 1.2270 |  |
|  | **IM** | Day 0 | 40.2453 | 0.1950 | 0.2103 |
|  |  | Day 7 | 40.7865 | 0.2842 |  |
|  | **P** | Day 0 | 31.8902 | 0.7626 | 0.0044 |
|  |  | Day 7 | 28.7672 | 2.1584 |  |
| **H3K4me3** | **I** | Day 0 | 9.3550 | 0.2071 | <.0001 |
|  |  | Day 7 | 6.1428 | 0.1323 |  |
|  | **IM** | Day 0 | 34.0199 | 0.0843 | <.0001 |
|  |  | Day 7 | 31.3955 | 0.0767 |  |
|  | **P** | Day 0 | 56.6229 | 0.3316 | <.0001 |
|  |  | Day 7 | 62.4680 | 0.2880 |  |
| **H3K9ac** | **I** | Day 0 | 15.5226 | 0.0815 | <.0001 |
|  |  | Day 7 | 9.9221 | 0.1791 |  |
|  | **IM** | Day 0 | 36.9021 | 0.0545 | 0.0041 |
|  |  | Day 7 | 35.3583 | 0.04633 |  |
|  | **P** | Day 0 | 48.5733 | 0.14805 | <.0001 |
|  |  | Day 7 | 54.7236 | 0.29051 |  |

**Supplementary Table 3.** Statistical data for analysis of centromere clustering in undifferentiated cells (day 0) and adipocytes (day 7). Data shows mean and standard deviation (SD) calculated for number of centromere clusters in nuclei.

| **Probe** | **Number of FISH signals on metaphase chromosomes** | **Number of analyzed nuclei** | **Day 0**  **(MSC)**  **Mean (**±**SD)** | **Day 7 (adipocytes)**  **Mean (**±**SD)** | **p-value** |
| --- | --- | --- | --- | --- | --- |
| **AC6** | 12 | 256 | 9.28 (± 1.88) | 9.17 (± 1.88) | 0.6283 |
| **SSCSR2** | 22 | 256 | 13.56 (± 3.13) | 19.56 (± 3.81) | < 0.0001 |
| **Cot-1 DNA** | 38 | 251 | 8.82 (± 2.68) | 24.34 (± 5.82) | < 0.0001 |
